# High Strength and Deformation Mechanisms of Al_0.3_CoCrFeNi High-Entropy Alloy Thin Films Fabricated by Magnetron Sputtering

**DOI:** 10.3390/e21020146

**Published:** 2019-02-04

**Authors:** Wei-Bing Liao, Hongti Zhang, Zhi-Yuan Liu, Pei-Feng Li, Jian-Jun Huang, Chun-Yan Yu, Yang Lu

**Affiliations:** 1 College of Physics and Optoelectronic Engineering, Shenzhen University, Shenzhen 518060, China; 2 Department of Mechanical Engineering, City University of Hong Kong, Hong Kong, China; 3 School of Physical Science and Technology, Shanghai Tech University, Shanghai 201210, China; 4 College of Mechatronics and Control Engineering, Shenzhen University, Shenzhen 518060, China; 5 College of Materials Science and Engineering, Shenzhen University, Shenzhen 518060, China; 6 Nano-Manufacturing Laboratory (NML), City University of Hong Kong Shenzhen Research Institute, Shenzhen 518057, China

**Keywords:** high-entropy alloys, thin films, hardness, deformation behaviors, nanocrystalline

## Abstract

Recently, high-entropy alloy thin films (HEATFs) with nanocrystalline structures and high hardness were developed by magnetron sputtering technique and have exciting potential to make small structure devices and precision instruments with sizes ranging from nanometers to micrometers. However, the strength and deformation mechanisms are still unclear. In this work, nanocrystalline Al_0.3_CoCrFeNi HEATFs with a thickness of ~4 μm were prepared. The microstructures of the thin films were comprehensively characterized, and the mechanical properties were systematically studied. It was found that the thin film was smooth, with a roughness of less than 5 nm. The chemical composition of the high entropy alloy thin film was homogeneous with a main single face-centered cubic (FCC) structure. Furthermore, it was observed that the hardness and the yield strength of the high-entropy alloy thin film was about three times that of the bulk samples, and the plastic deformation was inhomogeneous. Our results could provide an in-depth understanding of the mechanics and deformation mechanism for future design of nanocrystalline HEATFs with desired properties.

## 1. Introduction

It is well known that among all the alloy composition design systems, high-entropy alloys (HEAs) are a brand-new concept based on novel multi-component system composition designs. They contain at least four or five principal metal components and simply form a single face-centered cubic (FCC), body-centered cubic (BCC) or hexagonal close-packed (HCP) phase [1,2,3,4,5,6]. This novel concept is an important breakthrough of the past 25 years [7,8], as it is completely different from the traditional alloy design concepts in which one major component was selected, and other minor components were added to improve their related physical and chemical performances. It is worth mentioning that HEAs not only have simple phase structures, but also possess many excellent mechanical and physical properties, such as high tensile strength [9,10,11], good ductility at ambient and cryogenic temperatures [12,13], superior wear and fatigue resistance [14], and strong radiation tolerance [15,16]. These unique features qualify HEAs as potential engineering materials to meet the demanding requirements for complex and harsh environment applications, particularly in the turbine, aerospace, and nuclear industries [17,18,19,20,21]. However, the chemical composition of HEAs contains multiple elements which would naturally raise the cost for industrial application, and limit HEAs extensive development. As a consequence, to reduce the cost for future industrial applications and take full advantage of the above excellent comprehensive properties, HEA thin films (HEATFs) can be efficiently prepared and simultaneously coated on the surface of industrial components, especially for those complex geometry components. In these cases, the HEATFs will play an important role. The initial research of HEATFs is associated with the recent rapid development of HEAs and the high throughput preparation idea [22,23,24,25,26]. As the geometric size and microstructures of the thin films are different from the three-dimensional bulk samples, their performances under loading and service conditions could be completely different [27,28]. So far, HEATFs were verified to have remarkable effects on the hardness [24]. A series work on HEATFs was done not only on the high throughput fabrication but also on the mechanical properties, including the hardness and corrosion properties [29,30,31,32,33,34,35,36]. The previous work has greatly promoted the industrial application of HEATFs. Unfortunately, the related deformation behaviors have not been clearly revealed until now. To facilitate the use of HEATFs and provide a continuous coating technique, the deformation behaviors and reliability of HEATFs merit further investigation. Therefore, in this study we prepared the Al_0.3_CoCrFeNi HEATFs with a main simple FCC structure by magnetron sputtering, and fabricated nano-scaled pillars on the surface of the thin film by focus ion beams (FIBs), then utilized in situ scanning electron microscopy (SEM) compression to study the deformation behaviors of the HEATFs.

## 2. Materials and Methods

The target with a composition of Al_0.3_CoCrFeNi was prepared by metallurgy with high-purity (>99.99%) raw metal materials of aluminum, cobalt, chromium, iron, and nickel. The size of the target is ϕ76.2 × 3.175 mm. The Al_0.3_CoCrFeNi HEATFs were deposited on silicon wafer substrates by magnetron sputtering. Before putting the target in the vacuum chamber, it was cleaned by argon ion bombardment for about 2 min to remove the oxide or contaminants on the surface. To ensure a uniform deposition a rotation speed of the silicon wafer substrate was set at 2 rpm. The surface roughness of the as-deposited HEATFs was determined by white light interferometry (WLI) using Wyko NT9300 Surface Profiler (Veeco Instruments, Plainview, NY, USA), while the surface morphology and detail nanostructures were characterized by scanning electron microscopy (SEM) and atomic force microscopy (AFM) (Bruker Dimension Icon^TM^, Billerica, MA, USA) with ScanAsyst (Bruker Dimension Icon^TM^, Billerica, MA, USA) at room temperature. To investigate the phase structure of the as-deposited HEATFs, high-energy synchrotron radiation X-ray in transmission mode at 11-ID-C of Advanced Photon Source (APS) was used. The X-ray beam wavelength was 0.117418 Å. The detail microstructures of the as-deposited HEATFs were observed by high-resolution transmission electron microscopy (HRTEM) using a JEOL JEM-2100F instrument (JEOL, Akishima, Tokyo, Japan) operated at 200 kV. The chemical composition was analyzed by the energy dispersive X-ray spectrometer (EDS) equipped in the transmission electron microscopy (TEM). Nanoindentation experiments were performed using a Hysitron TI750 nanoindenter (Hysitron, Inc., Minneapolis, MN, USA) with a Berkovich tip. To avoid any potential effects of the substrate on the experiment, the indentation depth was kept to be less than 10% of the whole thickness of the HEATFs. Micropillars were fabricated out of the Al_0.3_CoCrFeNi HEAHFs by using a FEI Scios focused ion beam (FIB) (USA) (Thermo Scientific™, Hillsboro, OR, USA) at 30 kV/10pA as the final etching condition. The height of the nanopillars was kept to be less than the thickness of the Al_0.3_CoCrFeNi HEATFs. The in situ SEM compression tests were conducted at room temperature using a PI 85 PicoIndenter (Hysitron Inc.) with a flat punch diamond tip inside a FEI Quanta 450 FEG (USA) (Thermo Scientific™, Hillsboro, OR, USA), under displacement-control mode and at a strain rate of around 5×10^-3^ s^-1^. Raw load-displacement data were used to calculate the engineering stress and strain.

## 3. Results and Discussion

**Figure 1:**
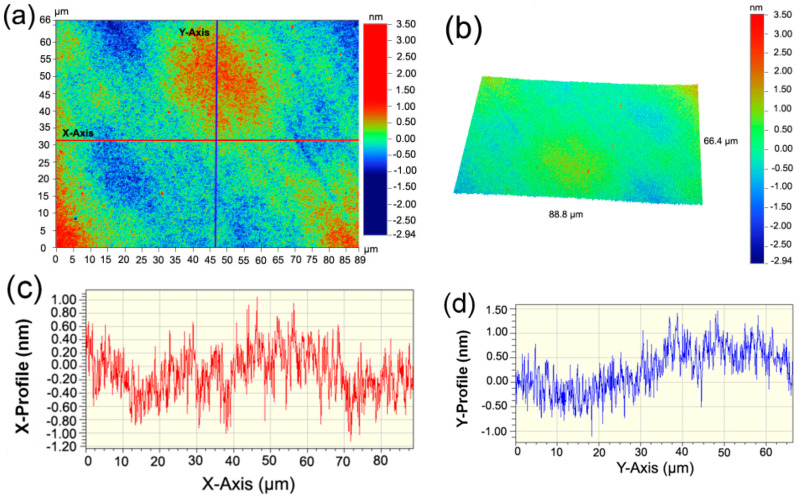
Surface profiles of the Al_0.3_CoCrFeNi high-entropy alloy thin films (HEATFs) characterized by white light interferometry (WLI) technique. (**a**) 2D surface profiles, (**b**) 3D surface profiles, (**c**,**d**): the profiles of the *x*-axis and *y*-axis as marked in (a) respectively.

Figure 1a,b show the two-dimensional (2D) and the three-dimensional (3D) surface roughness and profiles of the as-deposited Al_0.3_CoCrFeNi HEATFs, respectively, and Figure 1c,d show the X-profile and Y-profile of the corresponding positions selected on the HEATFs as marked in Figure 1a. It can be clearly seen that there are fine undulating nanostructures on the surface of the Al_0.3_CoCrFeNi HEATFs prepared by the magnetron sputtering deposition technique; however, the entire surface is very flat and smooth, with a roughness R_a_ of less than 3.5 nm.

**Figure 2:**
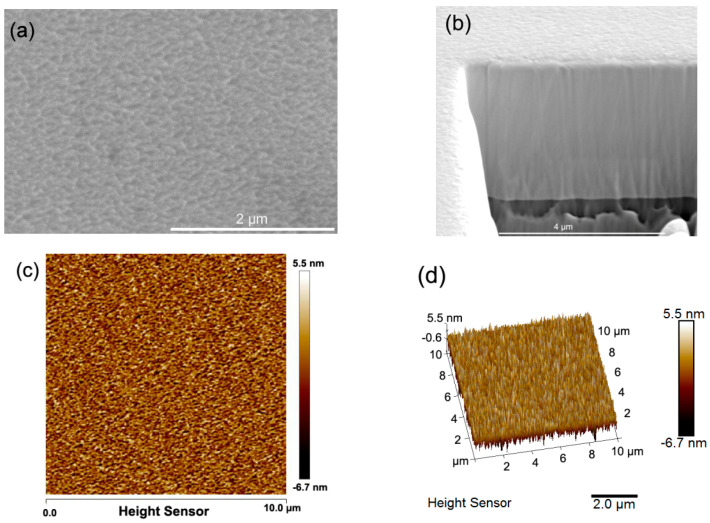
Surface morphologies and microstructures of the Al_0.3_CoCrFeNi HEATFs characterized in detail by scanning electron microscopy (SEM) and atomic force microscopy (AFM). (**a**) The SEM image of the surface morphologies, (**b**) the cross-section the HEATFs deposited on the silicon substrate, (**c**) the AFM image of the surface structure, and (**d**) 3D surface structures of the HEATFs.

The SEM surface morphology and the specific fine nanostructures of the as-deposited Al_0.3_CoCrFeNi HEATFs with a magnification of 50,000 times are shown in Figure 2a. It demonstrates that these fine nanostructures are well-knit and compact. The thickness of the HEATFs is about 4 μm, as shown in Figure 2b. To characterize the feature of the HEATFs in more detail, AFM scanning experiments were further conducted. Figure 2c,d show the 2D and 3D AFM images of the surface feature of the Al_0.3_CoCrFeNi HEATFs. Uniform nanostructures are clearly observed, and the heights of these undulating nanostructures were less than 5 nm, which is well consistent with that typically observed by the surface profile. All these experimental data verified that there were a lot of fine nanostructures on the surface of the Al_0.3_CoCrFeNi HEATFs, and the surface was very smooth as a whole, with a roughness R_a_ of less than 5 nm.

**Figure 3:**
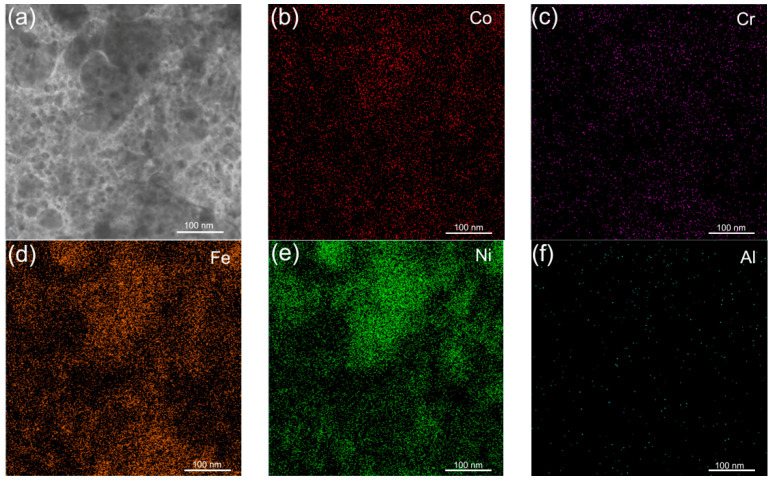
Element distribution mapping for the Al_0.3_CoCrFeNi HEATFs by TEM-EDS. The top TEM image shows the region analyzed. (**a**) Explanations for subfigure a; (**b**) explanations for subfigure b; (**c**) explanations for subfigure c; (**d**) explanations for subfigure d; (**e**) explanations for subfigure e; (**f**) explanations for subfigure f.

Figure 3a shows the TEM images and the corresponding EDS analysis of the Al_0.3_CoCrFeNi HEATFs. It can be seen that there are a lot of nanocrystalline structures in the HEATFs, with a grain size order of ~10 nm. The elemental distribution of the as-deposited HEATFs is homogenous. The bright and dark places shown in the TEM-EDS images are ascribed to the uneven sample thickness. Quantitative analysis by EDS confirms that the chemical composition is nearly the same as the composition of the sputtering target, as shown in Table 1.

**Figure 4:**
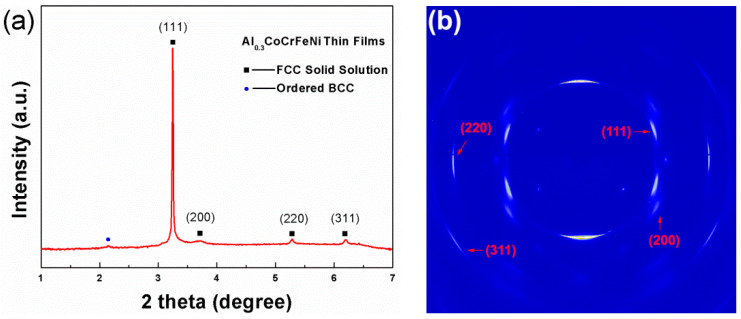
Phase structures of the HEATFs by high-energy synchrotron radiation X-ray. (**a**) synchrotron X-ray line profiles and (**b**) typical diffraction pattern.

To obtain the phase structural information of the Al_0.3_CoCrFeNi HEATFs, high-energy synchrotron radiation X-ray studies were undertaken. Figure 4a shows the X-ray line profiles of the HEATFs. The (111), (200), (220), and (311) phase peaks were observed and identified to be a typical FCC crystalline structure, whilst a small peak appeared before the (111) peak, which means that a minor ordered BCC NiAl type phase structure was in the HEATFs [37]. The corresponding diffraction patterns are exhibited in Figure 4b. The weak continuous rings certify that there are tiny polycrystalline structures in the HEATFs. Interestingly, the diffraction rings of the HEATFs were discrete, with obvious intensity differences, indicating that there were strong textures in the HEATFs. This event could be ascribed to the preferred growth of the thin film induced by the silicon substrate. It should be noted that a rigorous diffraction-intensity-distribution calculation of the solid solution phases responsible for certain orientations in the HEATFs is worthy of a focused topic. However, it is beyond the scope of this work. The synchrotron X-ray experimental results provide cogent evidence that the magnetron sputtering technique is an effect way to prepare the HEATFs with a simple phase structure. Moreover, it could also lead to a wide research range of HEAs, studying the corresponding properties from meso- to nanometer regimes.

**Figure 5:**
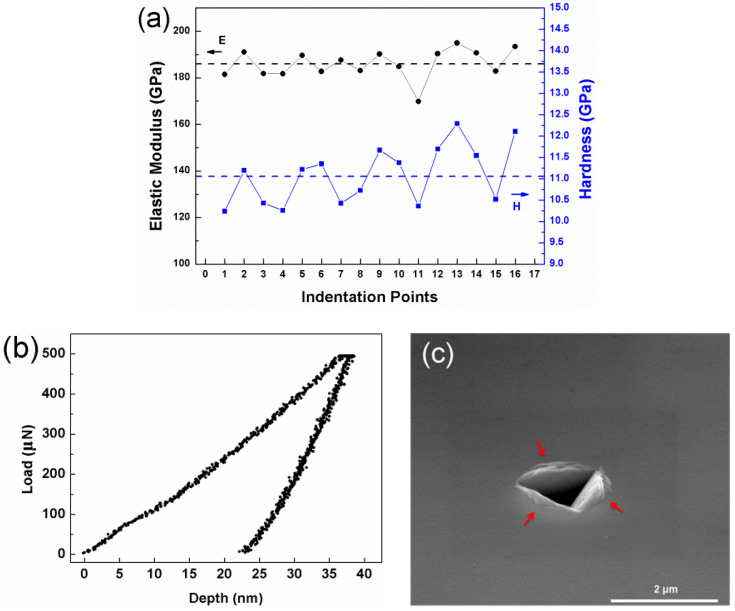
Nanoindentation properties of the as-deposited Al_0.3_CoCrFeNi HEATFs. (**a**) Elastic modulus and hardness of the HEATFs at different indentation points, (**b**) typical nano-indentation load–depth curve, and (**c**) typical SEM image of the impression mark.

Figure 5a shows the nanoindentation properties of the as-deposited HEATFs. Since a series of 4 × 4 matrix array indentation points were tested in sequence, the average values of elastic modulus and hardness were accurately calculated and were identified to be about 186.01 GPa and 11.09 GPa, respectively. It should be noted that the hardness of the HEATFs is about three times higher than that of the as-cast bulk Al_0.3_CoCrFeNi HEA sample, but the elastic modulus is nearly the same [38,39]. This enhanced hardness can be ascribed to the nanocrystalline strengthening mechanism which induced the hardening by a large number of grain boundaries observed in Figure 3a. Figure 5b is the typical nanoindentation load-depth curve of the HEATFs. It can be seen that as the loading force increases, the depth of the indenter pressed into the film gradually increases. After unloading, an irreversible depth was retained, indicating a plastic deformation has occurred on the surface of the HEATFs. Figure 5c exhibits the SEM image of the impression mark. The indentation profiles are self-similar. A remarkable pile-up (marked with red arrows) around the indentation can be clearly observed, suggesting that high localized plastic deformation occurred during nanoindentation.

**Figure 6:**
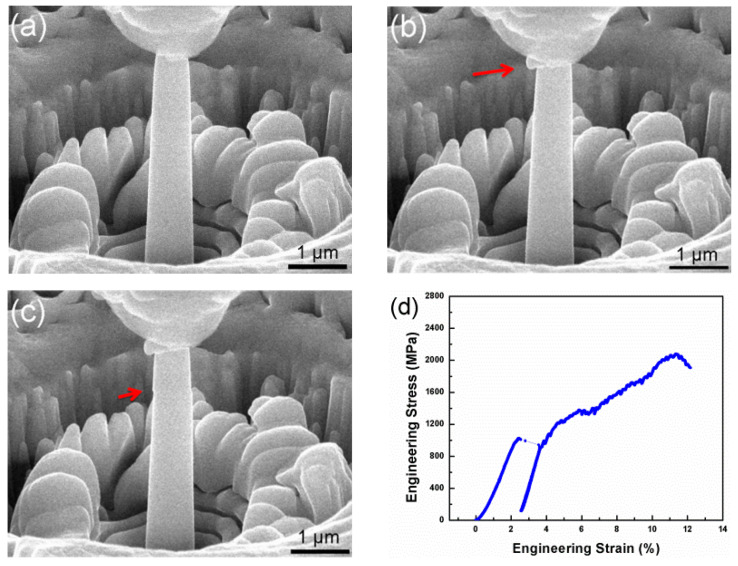
Compression properties for the Al_0.3_CoCrFeNi pillar with a diameter of 738 nm prepared from the HEATFs. (**a**) Elastic deformation stage, (**b**) localized plastic deformation occurred at the top of the pillar, (**c**) a slip generated at the top part of the pillar, (**d**) a typical compression engineering stress–strain curve of the pillar.

To further characterize the mechanical properties of the Al_0.3_CoCrFeNi HEATFs, a nanopillar sample with a diameter of 738 nm was fabricated from the HEATFs, and in situ SEM compression tests were conducted on the nanopillar sample, as shown in Figure 6. The entire compression deformation process of the nanopillar can be divided into the following stages. Initially, the nanopillar was deformed elastically, and no significant trace appeared on the surface of the nanopillar, as shown in Figure 6a. Secondly, with the increase of the compressive stress, a large localized metal flow and plastic deformation occurred at the top part of the nanopillar, as marked with a red arrow in Figure 6b. It indicates that the deformation of the Al_0.3_CoCrFeNi HEA nanopillar was inhomogeneous. After that, it can be observed that a slip was generated at the top part of the nanopillar, which was marked with a red arrow in Figure 6c. The occurrence of the slip is not only related to the plastic deformation, but also has an impact on the work hardening and the serration behavior of the HEAs [40]. The corresponding compression engineering stress–strain curve of the Al_0.3_CoCrFeNi HEA nanopillar is shown in Figure 6d. The yield strength of the nanopillar is about 1024 MPa, which is also about three times that of the bulk sample [38]. It is consistent with the above experimental results obtained by nanoindentation, as the strength is directly proportion with the hardness. After yielding the nanopillar exhibits work-hardening up to an ultrahigh strength. The compressive strength and corresponding strain were ~2075 MPa and ~11.39%, respectively. Following this, softening dominates until final fracture at a strain of ~12.14%. In general, the compression results further confirmed that the yield strength of the Al_0.3_CoCrFeNi HEATFs is about three times that of the bulk samples, and the plastic deformation is inhomogeneous.

## 4. Conclusions

In conclusion, the Al_0.3_CoCrFeNi HEATFs prepared by the magnetron sputtering technique were smooth, with a surface roughness R_a_ of less than ~5 nm. The chemical composition was homogeneous and amounts of nanocrystallines with a main single FCC phase structure formed in the HEATFs. The hardness of the HEATFs was ~11.09 GPa, and the yield strength of the nanopillar prepared from the HEATFs was ~1024 MPa. Both the hardness and the yield strength were about three times that of the bulk samples. Simultaneously, it was found that the plastic deformation of the HEATFs was inhomogeneous and localized. The present study could provide useful insights in the design and application of HEATFs for functional micro- and nano-devices.

## Figures and Tables

**Table 1:** The chemical composition of the as-deposited Al_0.3_CoCrFeNi HEATFs compared with that of the sputtering target.

Elements (at. %)	Al	Co	Cr	Fe	Ni
Nominal target	6.977	23.256	23.256	23.256	23.256
As-deposited HEATFs	6.452	23.436	24.602	23.623	21.887
